# Treatment of Rheumatism by Quinia and Veratria

**Published:** 1853-07

**Authors:** 


*Treatment of Rheumatism by Quinia and Veratria.*—The treatment of acute Rheumatism by Quinine, commenced by M. Briquet, and to a certain extent discontinued, on account of the doubtful character of the termination of one or two cases in which large quantities were administered, has been very much revived of late, and the impression respecting it gains favor daily. I have seen M. B. give it with good effect quite often. M. Valleix employs and recommends it at La Pitié, and I hope to be able to send a paper on the subject by a gentleman who attends his *Services* and Lectures. One gramme (15 grains) is administered once or twice a day, and the patient kept partly quininized. The gravity of the usual symptoms daily decreases, and it seems not to be at all hostile to cardiac complications. M. Trousseau, in commenting upon this and expressing his favorable impression, alluded also to the expenses attending the use of this agent, accompanying it at the same time with the relation of a remarkable instance of recovery from acute articular Rheumatism, which was then in his wards. It was true, he added, that it was as yet an isolated case, but the repetition of the means used might give equally favorable results. It followed the employment of Veratrine after the method recommended by M. Daniel (?). The patient, a woman under middle age, suffered intense pain in the articulations, high fever with endocarditis exhibiting itself by a derangement of the first sound at the base of the heart—(sigmoid valves of aorta)—with a souffle prolonged into the large vessels, and accompanied with an alteration in the left ventricle also. In sixty-two hours she was absolutely cured, leaving not the slightest trace of febrile excitement, the presence of a slight souffle alone remains. One half of a milligramme (about 1-10th of a grain?) was given in the form of pill, each day increasing the quantity, and, when the pains were relieved, continuing it in the same dose every second day. Its influence on the circulation in this case was prodigious, to use M. Trousseau's expression; the pulse fell to 42, and when I heard of the patient two days after, it was still at this reduced rate. Veratrine, we are aware, is one of the active principles of colchicum, and it is, therefore, not surprising that it should possess some power in acute Rheumatism, more particularly in those dependent upon the arthritic diathesis, gout, etc.—*Ibid.*

